# Supplementary material for: Gene age shapes the transcriptional landscape of sexual morphogenesis in mushroom-forming fungi (Agaricomycetes)
Source: eLife. 2022 Feb 14;11:e71348. doi: 10.7554/eLife.71348 (PMC8893723; doi:10.7554/eLife.71348)
Supplement: Transparent reporting form [file elife-71348-transrepform1.docx]

***eLife’s* transparent reporting form**

We encourage authors to provide detailed information *within their submission* to facilitate the interpretation and replication of experiments. Authors can upload supporting documentation to indicate the use of appropriate reporting guidelines for health-related research (see [EQUATOR Network](http://www.equator-network.org/%20)), life science research (see the [BioSharing Information Resource](https://biosharing.org/)), or the [ARRIVE guidelines](http://www.plosbiology.org/article/info:doi/10.1371/journal.pbio.1000412) for reporting work involving animal research. Where applicable, authors should refer to any relevant reporting standards documents in this form.

If you have any questions, please consult our Journal Policies and/or contact us: [editorial@elifesciences.org](mailto:editorial@elifesciences.org).

**Sample-size estimation**

- You should state whether an appropriate sample size was computed when the study was being designed
- You should state the statistical method of sample size computation and any required assumptions
- If no explicit power analysis was used, you should describe how you decided what sample (replicate) size (number) to use

Please outline where this information can be found within the submission (e.g., sections or figure legends), or explain why this information doesn’t apply to your submission:

*In this study, two species were sampled for developmental transcriptomic analysis. No explicit prior power analysis was carried out. Instead, we took the experimental layouts of several published developmental transcriptome studies from the literature as examples, where 3-5 samples from fruiting body were commonly taken.*

*Pterula* *gracilis, with a very simple morphology, was sampled four times during fruiting body formation, while* *Pleurotus ostreatus which is a pileate-stipitate species was sampled at seven developmental stages. Additionally, we sampled different tissue types, two types in two stage (P3 and YFB) and four types in the mature fruiting body stage.*

*We excluded one developmental stages (stage 2 primordia) from the analysis, due to the high variation among biological replicates.*

**Replicates**

- You should report how often each experiment was performed
- You should include a definition of biological versus technical replication
- The data obtained should be provided and sufficient information should be provided to indicate the number of independent biological and/or technical replicates
- If you encountered any outliers, you should describe how these were handled
- Criteria for exclusion/inclusion of data should be clearly stated
- High-throughput sequence data should be uploaded before submission, with a private link for reviewers provided (these are available from both GEO and ArrayExpress)

Please outline where this information can be found within the submission (e.g., sections or figure legends), or explain why this information doesn’t apply to your submission:

*For each sample type, tissue from 3-8 individual fruiting bodies was pooled into a biological replicate. Three biological replicates were sampled from all developmental stages and tissue types for RNA-Seq. The concordance of replicates was checked with MDS analysis and hierarchical clustering. Technical replicates were not included in the study. Raw data have been deposited to NCBI’s GEO archive (GSE176181). In case of Pleurotus the biological replicates of stage 2 primordia showed high variability according to the MDS analysis, therefore we omitted all replicates of this stage from all further analysis.*

**Statistical reporting**

- Statistical analysis methods should be described and justified
- Raw data should be presented in figures whenever informative to do so (typically when N per group is less than 10)
- For each experiment, you should identify the statistical tests used, exact values of N, definitions of center, methods of multiple test correction, and dispersion and precision measures (e.g., mean, median, SD, SEM, confidence intervals; and, for the major substantive results, a measure of effect size (e.g., Pearson's r, Cohen's d)
- Report exact p-values wherever possible alongside the summary statistics and 95% confidence intervals. These should be reported for all key questions and not only when the p-value is less than 0.05.

Please outline where this information can be found within the submission (e.g., sections or figure legends), or explain why this information doesn’t apply to your submission:

*The used statistical analysis can be found in the Materials and Methods part of the manuscript. Exact p-values, additional values (e.g. odds-ratios) and the name of statistical analysis were indicated in the figures, tables and the Results and Discussion part of the manuscript.*

(For large datasets, or papers with a very large number of statistical tests, you may upload a single table file with tests, Ns, etc., with reference to sections in the manuscript.)

**Group allocation**

- Indicate how samples were allocated into experimental groups (in the case of clinical studies, please specify allocation to treatment method); if randomization was used, please also state if restricted randomization was applied
- Indicate if masking was used during group allocation, data collection and/or data analysis

Please outline where this information can be found within the submission (e.g., sections or figure legends), or explain why this information doesn’t apply to your submission:

*Due to the nature of our analysis this point is not relevant.*

**Additional data files (“source data”)**

- We encourage you to upload relevant additional data files, such as numerical data that are represented as a graph in a figure, or as a summary table
- Where provided, these should be in the most useful format, and they can be uploaded as “Source data” files linked to a main figure or table
- Include model definition files including the full list of parameters used
- Include code used for data analysis (e.g., R, MatLab)
- Avoid stating that data files are “available upon request”

Please indicate the figures or tables for which source data files have been provided:

*Raw RNA-Seq reads have been deposited to NCBI’s GEO archive (GSE176181).*

*Other files associated with this paper (including species tree, code used for data analysis) have been deposited in Dryad (private link:* [*https://datadryad.org/stash/share/xl6SMTywZzaJg_92vnO7btOOBRTWddpZwr7dGLZtgjw*](https://datadryad.org/stash/share/xl6SMTywZzaJg_92vnO7btOOBRTWddpZwr7dGLZtgjw) *final availability:* [*https://doi.org/**10.5061/dryad.5qfttdz5m*](https://doi.org/10.5061/dryad.5qfttdz5m) *).*
